# Pandemic Best Regulatory Practices: An Urgent Need in the COVID‐19 Pandemic

**DOI:** 10.1002/cpt.1932

**Published:** 2020-07-04

**Authors:** Murray M. Lumpkin, John C. W. Lim

**Affiliations:** ^1^ The Bill and Melinda Gates Foundation Seattle Washington USA; ^2^ Centre of Regulatory Excellence Duke-NUS Medical School Singapore; ^3^ SingHealth Duke-NUS Global Health Institute Singapore

## Abstract

As large numbers of candidate drugs and vaccines for potential use in the coronavirus disease 2019 (COVID‐19) pandemic are being investigated, medicine regulators globally must now make urgent, informed, contextually risk‐1based decisions regarding clinical trials and marketing authorizations. They must do this with the flexibility demanded by the pandemic while maintaining their core risk assessment and public safety functions. We lay out the critical role of regulators in the current crisis and offer eight “pandemic best regulatory practices.” These should support both the regulatory public heath imperative and assure timely patient access to effective, safe, quality products worldwide during this emergency—thus contributing to ending this pandemic as quickly, effectively, and safely as possible.

The coronavirus disease 2019 (COVID‐19) pandemic is different in many ways from other recent public health emergencies, especially in its massive global impact on human life and economic activities. Researchers globally are working tirelessly to develop therapeutics, vaccines, diagnostics, protective equipment, and other items needed to control the pandemic. There is pressure on the world’s leading national regulatory authorities (NRAs) for medicines and medical devices to take urgent, informed, contextually risk‐based decisions regarding clinical trials authorizations, emergency use authorizations, site inspections, and postauthorization commitments. This, in turn, creates pressure for similar actions in many other countries, especially those whose NRAs are significantly under‐resourced.

Whereas developing effective therapeutics and vaccines is the immediate challenge, once they are available, an equally complex and formidable challenge will be responding to immediate global demand for equitable access to these products in terms of cost and time. Assuring almost simultaneous global access to quality versions of these products will require new approaches in many aspects of the chain of events (viz. regulatory, utilization policy, procurement, and delivery) that leads ultimately to patient access. New product roll‐out only to a few countries or gradual roll‐out across the globe will not be acceptable.

The regulatory component is a vital link in the event chain ensuring patient access to quality therapies in an emergency.

The work of NRAs is often overlooked, underappreciated, and even maligned. We have, however, learned in other public health emergencies that without *interpretable* data to guide their actions, the heroic efforts of first responders and medical personnel are severely hampered. This has already been experienced to varying degrees in the initial stages of the current pandemic. Eichler *et al*. described recently the current clinical and ethical challenges of large numbers of uncoordinated COVID‐19 clinical trials, which, by design and conduct, are destined to be non‐informative.^1^ The only thing worse than no diagnostic or no treatment is an unreliable diagnostic or an uninformed treatment. Millions of people are placing their hopes on new medical interventions, and they deserve better than nonscientific hunches. It is an inconvenient truth: we cannot simply “wish” these products to work.

The role of regulators—particularly critical in emergencies—is to assure that data about needed products are *transparent*, *interpretable*, and* reliable*. This enables practitioners, patients, families, and governments to make truly informed decisions. For regulators to perform this vital role in a pandemic effectively requires *flexibility*. This is not flexibility in applying fundamental scientific principles, but rather in how regulators work together to minimize redundancy and time loss, thus maximizing the available human, scientific, and financial regulatory resources, which are vastly constrained internationally.

Whereas effective medical interventions are currently being developed, NRAs must now simultaneously work to prepare for rapid global regulatory actions once interpretable data are developed in order to help expedite patient access to safe, good quality, and reliable diagnostics and effective therapies.

Due to the work of many regulators and organizations over the past few decades,^2^, ^3^, ^4^, ^5^ there are already established pathways through which regulators work collaboratively and have aligned on many basic approaches to the performance of their responsibilities. These regulatory harmonization, convergence, alignment, and reliance initiatives have been a prescient preparation for this moment in history. If not now, when?

Many countries and the World Health Organization (WHO) have established regulatory pathways for handling emergencies.^6^, ^7^, ^8^ We do not need to reinvent the regulatory wheel in the middle of this worldwide crisis. We need to use these established emergency pathways transparently, confidently, and globally.

To ensure that the regulatory steps in the patient access chain are implemented efficiently and effectively in the current situation, a “*pandemic best regulatory practices*” approach should be used. These must be immediately actionable practices that have the support of industry, regulators, and governments.

As our knowledge of this pandemic evolves, we will continually learn what should be added to these *pandemic best regulatory practices*. Some of these may not be feasible under routine agency practices, but a pandemic is not routine. Most governments have provisions to allow special procedures during emergencies to meet extraordinary needs without compromising fundamental aspects of science or product quality.

The practices described below will help NRAs most efficiently and effectively reference and utilize the actions and work products of other regulatory authorities they trust to help inform their own decisions. This will help them make timely, well‐informed, efficient, appropriate, and scientifically robust public health decisions for their jurisdictions during this pandemic.

## PANDEMIC BEST REGULATORY PRACTICES

1. *Regulatory authorities should rely wherever possible on the actions taken by and the regulatory work products of trusted NRAs and the WHO prequalification unit to inform their own decisions*. To do so efficiently, NRAs and the WHO should share full, unredacted scientific assessment reports and inspection reports electronically with other regulatory agencies. Given the gravity of the current situation and limitations on travel, the need for such “regulation through reliance” on the work products of trusted agencies to assure efficient, yet scientifically robust, assessments requires full reports be made available immediately either on demand or, better, on an agency website for ease of access by other regulators. The default position should be that companies concur with this process and will not raise “confidentiality” or “trade secret” arguments that result in time-consuming redactions and documents with inadequate information for the receiving agency. Uninformative reports compel duplication of assessments already performed by other agencies, which, in the context of a pandemic, leads to an unconscionable waste of time and resources. As highlighted in one of the recommendations of a recent US National Academies of Sciences, Engineering and Medicine consensus study report, with access to such full reports, NRAs are better positioned to determine more efficiently and robustly if a product is appropriate for their health care systems and populations.^9^ This is especially true now in the context of the pandemic.
2. *During a pandemic, a product should be labeled on the carton only with the international nonproprietary name, the manufacturer, the site of manufacture, and the dosage form and amount*. In addition, there should be a Quick Response code (*QR code*) that links directly to all authorized package labeling and summary of product characteristics/package inserts and patient leaflets for all countries where the product is either fully or emergency authorized. This should generally be hosted on the manufacturing company’s website. One should be able to read the authorized labeling by scrolling to the labeling authorized in a specific country. If a hard copy is needed, printing would be easily done on site. In addition, there is assurance of immediate access to the most recent version of the authorized labeling, which may be changing rapidly with increasing knowledge of how the product should be used in the pandemic. This would also facilitate transfer of the product from country to country without having to relabel, thus saving valuable time, human and financial resources, and would allow a scarce product to be used where it is needed most rapidly. This would be especially helpful for frozen vaccines, as it would mean products would not have to be thawed for re-labeling if they were going to be used in a country different from where the original label was intended.
3. *The standard pandemic marketing application dossier should be submitted in International Council for Harmonisation of Technical Requirements for Pharmaceuticals for Human Use (ICH) electronic Common Technical Dossier format,* ^10^ or hardcopy, if necessary, and should be accepted in that format by all jurisdictions without any local or regional adaptations, other than language and local specifics (Module 1).
4. *The first NRAs to receive and review a marketing authorization dossier, or request for emergency authorization for a pandemic product (e.g*.,* European Medicines Agency Committee on Human Medicinal Products meeting, US Food and Drug Administration advisory committee or internal meeting,* and* Swissmedic internal discussion), should routinely open the discussion for participation or observation via teleconferencing to the* *WHO prequalification assessors and other NRAs*. The discussion and reasons for the action taken by the initial authority will then be better understood and can be more quickly incorporated into the decision making of these other agencies. Companies should not assert confidentiality or trade secret reasons for limiting such access by global regulators and the WHO prequalification assessors, given the pandemic situation.
5. *When the pandemic product is subsequently offered to a country, the applicant must make it* *clear which version of the product is being presented and if it is the same version as has been authorized by the trusted reference agency*. Any differences in the manufacturing process and/or site and other version differences should be highlighted and explained in the initial cover sheet of the documentation presented to the receiving NRA. Many versions of a new product will quickly appear, both legitimate and, sadly, some that are substandard or falsified. NRAs must assure that a product in its jurisdiction is what it is labeled to be and that it will perform as expected. This means assurance of compliance with international standards of quality manufacturing and evidence of reliable performance in patients if it is a different version from that assessed by the initial reference NRA.
6. *Provided the same version is being sent to the receiving country*,* as assessed by the reference NRA, further local batch release testing or other laboratory testing of the product should not routinely be required during a pandemic*.
7. *Local clinical efficacy and safety trials should not routinely be required for authorization during a pandemic, unless there is a strong scientific argument that the data in the dossier are not extrapolatable to the local population or health care system*. If local trials are necessary to assure local population specific questions are addressed, these should be designed and powered to adequately address the questions raised.
8. *Given the relatively constrained amount of data available at the time of an emergency authorization, postauthorization safety and efficacy surveillance will be of paramount importance to the global community*. Any reports of suspected product lack of efficacy or product toxicity must be reported transparently and immediately to the manufacturer (if known) and the NRA. NRAs should report immediately to the WHO Uppsala Monitoring Centre so that global reports can be consolidated and assessed, and any new critical information disseminated transparently, quickly, and globally. Especially given concerns about product availability and equitable access, postauthorization monitoring should also focus on potential substandard and falsified versions of authorized products. These should be reported to the WHO Global Surveillance and Monitoring Systems for maximum visibility and quick information dissemination to regulators globally.

We have defined this initial list of *pandemic best regulatory practices* to prompt NRAs globally to review their readiness and processes to expedite sound and scientifically robust regulatory reviews of therapeutic products emerging during the current pandemic. We recommend that the WHO should maintain such an evolving list on its website and advocate strongly for their implementation.

Using these and other additional *pandemic best regulatory practices* will facilitate global regulatory excellence in helping assure timely patient access to effective, safe, quality products worldwide, and thus contribute to ending this pandemic as quickly, effectively, and safely as possible.

## Funding

No funding was received for this work.

## Conflict of interest

The authors declared no competing interests for this work.

## Disclaimer

The views expressed in this article are the personal views of the author(s) and may not be understood or quoted as being made on behalf of or reflecting the position of the organizations at which the author(s) is/are employed/affiliated.
